# Chronic maternal hepatitis B virus infection and pregnancy outcome- a single center study in Kunming, China

**DOI:** 10.1186/s12879-021-05946-7

**Published:** 2021-03-10

**Authors:** Qian Sun, Terence T. Lao, Mingyu Du, Min Xie, Yonghu Sun, Bing Bai, Junnan Ma, Tianying Zhu, Shengnan Yu, Runmei Ma

**Affiliations:** 1 Department of Obstetrics and Gynecology, the First Affiliated Hospital of Kunming Medical University, PO box 650032, No.295, Xi Chang Road, Kunming, Yunnan China; 2 Department of Obstetrics and Gynecology, the Chinese University of Hong Kong, Hong Kong, China; 3 Department of Obstetrics and Gynecology, Kunming Angel Women and Children’s Hospital, PO box 650108, No 199, Dianmian Road, Kunming, Yunnan China

**Keywords:** Chinese, Maternal hepatitis B virus, Infection, Prevalence, Pregnancy outcome

## Abstract

**Background:**

Chinese population has a high prevalence of chronic hepatitis B virus (HBV) infection, the impact of which on pregnancy outcome remains controversial. A single-center retrospective cohort study was performed in Kunming, a multi-ethnic city in south-western China to examine this issue.

**Methods:**

The singleton pregnancies delivering at ≥28 weeks gestation under our care in 2005–2017 constituted the study cohort. Maternal characteristics and pregnancy outcome were compared between mothers with and without seropositivity for hepatitis B surface antigen (HBsAg) determined at routine antenatal screening.

**Results:**

Among the 49,479 gravidae in the cohort, the 1624 (3.3%) HBsAg seropositive gravidae had a lower incidence of nulliparity (RR 0.963, 95% CI 0.935–0.992) and having received tertiary education (RR 0.829, 95% CI 0.784–0.827). There was no significant difference in the medical history, pregnancy complications, or labor or perinatal outcome, except that HBV carriers had significantly lower incidence of labor induction (RR 0.827, 95% CI 0.714–0.958) and of small-for-gestational age (SGA) infants (RR 0.854, 95% CI 0.734–0.994). On regression analysis, maternal HBV carriage was independently associated with spontaneous labor (aRR 1.231, 95% CI 1.044–1.451) and reduced SGA infants (aRR 0.842, 95% CI 0.712–0.997).

**Conclusions:**

Our 3.3% prevalence of maternal HBV infection was around the lower range determined in the Chinese population. The association with spontaneous labor and reduced SGA infants could have helped to promote the perpetuation of the infection through enhanced survival of the offspring infected at birth, thus explaining the high prevalence in the Chinese population.

## Background

Chronic hepatitis B virus (HBV) infection is probably the commonest chronic viral infections globally, as it can be found in up to 20% of the populations in parts of Africa, the Mediterranean basin, Eastern Europe, and particularly the Asia-Pacific region [1–3]. The success of HBV as an infectious agent is most likely related to an ability to co-evolve within its human host since the migration of humans out of Africa 100,000 years ago, co-expanding and co-migrating with the human populations to cumulate in the HBV pandemic that correlated with the global population increase over the last 5000 years [4–6]. In regions with high prevalence of HBV infection, vertical transmission is the major mode of infection and is responsible for the maintenance of the high endemic city in these regions [7]. If maternal HBV infection exerts adverse effects on the pregnancy, this could impact offspring survival hence reducing the likelihood of perpetuating the infection down successive human generations. So the vital question is whether maternal HBV infection could be detrimental to obstetric outcome?

In the literature, the obstetric effects of maternal chronic HBV infection remains conflicting. A number of studies have found no or minimal adverse pregnancy outcomes [8–12]. Indeed, there is even a suggestion that maternal HBV infection is associated with enhanced fetal growth, as reduced small-for-gestational age (SGA) infants [9, 12], and increased birthweight and incidence of large-for-gestational age (LGA) as well as macrosomic infants [13–15] have been reported. These observations would be compatible with the notion that chronic HBV infection has co-evolved with humans without jeopardizing pregnancy outcome so as to ensure transmission to successive generations. On the other hand, there are also studies which found associations with preterm birth (PTB) [10, 13, 16–21], pregnancy hypertensive disorders (PHD) [13, 15], gestational diabetes mellitus (GDM) [22–25], low-birthweight (LBW) infants [17, 26, 27], asphyxia [28], and intrauterine fetal death (IUFD) and stillbirth (SB) [13, 17]. The reason(s) for the conflicting and contradicting observations remains unclear, but as these reports were based on different racial/ethnic groups in different locations, one possible factor could have been racial/ethnic differences among the different studies.

China has a vast population and is endemic with HBV infection, where the latest review and meta-analysis on HBV infection found the prevalence in the general population of 5 to 7.99%, of which more than 90% of the infected subjects are older than 20 years [29]. Yunnan is a province in the mountainous south-western China where the residents consist of at least 22 different ethnic groups, including some ethnic minority groups which are unique to the province, and the region is much less developed and affluent compared with most of the other Chinese provinces. Little is known about the prevalence and impact of maternal HBV infection on pregnancy outcome in this multi-ethnic area in China. We therefore conducted this single centre cohort study to elucidate the relationship between maternal HBV infection with obstetric outcome in the city of Kunming, China, to examine this issue.

## Materials and methods

This was a retrospective study based on data from the Medical Birth Register for the period January 2005 to December 2017 in the First Affiliated Hospital of Kunming Medical University, a tertiary referral hospital located in the provincial capital of Yunnan province, China. After exclusion of women with stillbirth, birth < 28 weeks of gestation (which was regarded as miscarriage in China and no active resuscitation would be provided) and pregnancy termination for aneuploidy or major defects, and multifetal pregnancies, there were 49,479 pregnancies in the final analysis.

Baseline characteristics, obstetric history including previous miscarriage, abortion (pregnancy termination) and cesarean section (CS), and pregnancy/labor complications and outcomes were collected from the Medical Birth Register. Information on the mode of conception of the index pregnancy as either spontaneous or through assisted reproduction technology (ART) was also elicited. Maternal age referred to age at delivery and the age of 35 years or older was used to categorize mothers as those of advanced maternal age (AMA). Level of education was dichotomized as tertiary or below. Gestational age was determined based on either date of last menstrual period or ultrasound examination in the first trimester. Data on height and weight before pregnancy were collected at the first antenatal visit, and pre-pregnancy body mass index (BMI) was calculated as (weight in kg)/ (height in meter)^2^, with further classification of the parturients into overweight (≥24 kg/ m^2^) / obese (≥28 kg/m^2^) according to the standard of Working Group on Obesity in China [30]. Maternal pre-existing medical conditions analysed include chronic hypertension (hypertension diagnosed prior to conception or found at the first antenatal examination) and pre-gestational diabetes (diabetes diagnosed prior to pregnancy irrespective of type of treatment). The pregnancy complications and outcomes examined are PHD (including gestational hypertension and preeclampsia), GDM, placenta previa, placental abruption, PTB at < 34 and at 34 to < 37 completed weeks of gestation, post-dated pregnancy (delivery at/later than 10 days after the due date or at a gestational age ≥ 41.4 weeks), labor induction and CS (inclusive of elective and emergency CS), postpartum hemorrhage (PPH, defined as blood loss of ≥500 ml for vaginal delivery and ≥ 1000 ml for CS), maternal admission to intensive care unit (ICU), and maternal mortality within 42 days of delivery. The diagnosis of diabetes mellitus complicating pregnancy was according to WHO criteria [31] before the year of 2014, and IADPSG criteria [32] since 1st January, 2014. The diagnosis of hypertensive disorders in pregnancy was made according to the guidelines of the International Society for the Study of Hypertension in Pregnancy [33]. Infant outcome examined included SGA and LGA infants, admission to neonatal unit (NNU) for both observation and treatment, and neonatal mortality (within 7 days of birth). The definitions of SGA and LGA were neonates with birth weight below the 10th percentile or above the 90th percentile for gestation respectively, according to the local data of birth weight in Kunming [34]. These characteristics and outcomes were compared between pregnancies in mothers seropositive versus seronegative for HBsAg.

For statistical analysis, continuous variables were presented as mean ± standard deviation (SD) and categoric variables as percentages (%). Univariate analysis was performed using t test and χ2 test for continuous and categorical variables respectively. Logistic regression analysis was used to examine the association between HBsAg seropositivity with pregnancy outcomes. In the first model, adjustment was made for the effects of nulliparity status, AMA, high BMI, previous miscarriage, abortion and CS. In the second model, in addition to the above adjustments, further adjustments were made for history of tertiary education and pregnancy through ART technology. The results are expressed as relative risk (RR) and adjusted relative risk (aRR) with their respective 95% confidence intervals (95%CI). The statistical software package SPSS Statistics 22.0 (SPSS Inc., Chicago, IL, USA) was used for data analyses, and *P* < 0.05 was regarded as statistically significant.

## Results

Among the 49,479 women in the database, 1624 (3.3%) were screened HBsAg seropositive. There were no difference in mean maternal age, incidence of AMA, height, weight, BMI or overweight gravidae between the HBsAg seropositive and seronegative women, but the HBsAg seropositive women had a slightly but significantly higher mean gestational weight gain, a slightly but significantly lower incidence of nulliparity (RR 0.963, 95% CI 0.935–0.992), and women who received tertiary education (RR 0.829, 95% CI 0.784–0.827, Table 1).

**Table 1:** Maternal characteristics between HBV carriers and non-carriers with singleton pregnancies delivering at/beyond 28 completed weeks of gestation

	Hepatitis B carrier			
	Yes (*n* = 1624)	No (*n* = 47,855)	*p*	RR (95% CI)
Age (years)	29.6 ± 4.4	29.7 ± 4.3	0.666	–
≥35 years (%)	13.2	13.6	0.653	0.971 (0.856–1.103)
Height (cm)	159.9 ± 4.9	160.1 ± 5.3	0.232	–
Weight pre-pregnant (kg)	53.2 ± 8.0	53.5 ± 8.0	0.179	–
Weight pre-delivery (kg)	67.8 ± 8.4	67.7 ± 8.7	0.584	–
Gestational weight gain (kg)	14.5 ± 4.9	14.3 ± 4.9	0.030	–
Body mass index (kg/m^2^)	20.8 ± 3.0	20.8 ± 2.9	0.585	–
Overweight/obese (%)	12.6	12.8	0.846	0.987 (0.867–1.124)
Nulliparity (%)	73.1	76.1	0.009	0.963 (0.935–0.992)
Previous miscarriage ≥1 (%)	1.3	1.0	0.296	1.260 (0.817–1.945)
Previous abortion ≥1 (%)	12.9	12.5	0.574	1.038 (0.913–1.183)
Previous cesarean delivery (%)^a^	42.4	45.8	0.168	0.927 (0.829–1.036)
Pregnancy by ART (%)	1.2	0.7	0.045	1.595 (1.008–2.524)
Tertiary education (%)	43.3	52.2	< 0.001	0.829 (0.784–0.827)

^a^Among multiparous gravidae

When the pregnancy outcomes were compared between the two groups, no difference in the incidence of either chronic hypertension or pre-gestational diabetes, or in pregnancy complications, could be found (Table 2). For PHD, the two groups had similar incidences whether for overall incidence of pregnancy hypertensive disorders, gestational hypertension alone, or preeclampsia alone. Similarly for PTB, no difference in overall PTB, or in early (< 34 weeks gestation) or late PTB could be demonstrated.

**Table 2:** Pregnancy outcome between HBV carriers and non-carriers with singleton pregnancies delivering at/beyond 28 completed weeks of gestation

	Hepatitis B carrier			
	Yes (*n* = 1624)	No (*n* = 47,855)	*p*	RR (95% CI)
Chronic hypertension (%)	0.4	0.4	0.709	1.154 (0.543–2.451)
All hypertensive pregnancy (%)	4.2	4.6	0.442	0.912 (0.720–1.155)
Gestational hypertension (%)	1.6	1.9	0.368	0.835 (0.563–1.238)
Pre-eclampsia/eclampsia (%)	2.7	2.8	0.788	0.960 (0.711–1.295)
Pre-gestational diabetes (%)	0.6	0.6	0.881	.951 (0.490–1.843)
Gestational diabetes (%)	13.9	15.5	0.073	0.895 (0.791–1.012)
Placenta previa (%)	2.2	2.9	0.126	0.775 (0.559–1.076)
Placental abruption (%)	0.8	0.9	0.802	0.932 (0.538–1.615)
Preterm birth < 34 weeks (%)	2.0	2.2	0.577	0.904 (0.635–1.288)
Preterm birth 34- < 37 weeks (%)	6.3	5.8	0.437	1.080 (0.890–1.310)
All preterm birth (%)	8.1	7.8	0.706	1.033 (0.874–1.220)
Postdated (≥41.4 weeks) birth (%)	0.4	0.3	0.845	1.085 (0.481–2.446)

For labor/delivery outcome, women seropositive for HBsAg had significantly lower incidence of labor induction (RR 0.827, 95% CI 0.714–0.958), but no difference in incidence of CS, PPH overall or severe PPH, or admission into the ICU (Table 3). For infant outcome, there was no difference in the mean gestational age at delivery or birthweight, incidence of LGA infants, admission into the NICU, or neonatal death, but there was a small but significantly lower incidence of SGA infants (RR 0.854, 95% CI 0.734–0.994).

**Table 3:** Labor / delivery and maternal and infant outcome between HBV carriers and non-carriers with singleton pregnancies delivering at/beyond 28 completed weeks of gestation

	Hepatitis B carrier			
	Yes (*n* = 1624)	No (*n* = 47,855)	*p*	RR (95% CI)
Labor induction (%)	10.1	12.2	0.010	0.827 (0.714–0.958)
Cesareran delivery (%)	34.9	34.8	0.949	1.002 (0.937–1.072)
All postpartum hemorrhage (%)	10.7	10.3	0.667	1.032 (0.894–1.192)
Postpartum hemorrhage ≥1500 ml (%)	1.0	1.1	0.647	0.891 (0.543–1.462)
Matertnal ICU admission (%)	0.7	0.6	0.630	1.152 (0.648–2.046)
Maternal death (%)	0 (*n* = 0)	0 (*n* = 9)	1.000	–
Gestational age (weeks)	38.9 ± 1.6	38.9 ± 1.9	0.956	–
Birthweight (g)	3172 ± 459	3158 ± 534	0.254	–
Large-for-age (%)	8.4	7.9	0.445	1.066 (0.905–1.255)
Small-for-age (%)	9.5	11.2	0.040	0.854 (0.734–0.994)
Admit NICU (%)	17.2	17.3	0.924	0.995 (0.892–1.109)
Neonatal death (%)	0.4	0.5	0.776	0.897 (0.423–1.899)

Finally multiple logistic regression analysis was performed to examine the independent association between HBsAg seropositivity with various pregnancy outcomes (Table 4). In the first model, the confounding variables adjusted for included nulliparity status, AMA, high BMI, previous miscarriage, abortion and CS. Maternal HBsAg seropositivity was independently associated with increased spontaneous labor (aRR 1.231, 95% CI 1.044–1.451) and reduced SGA infants (aRR 0.842, 95% CI 0.712–0.997). When additional adjustment was made for the confounding factors of tertiary education and pregnancy by ART technology (model two), the associations remained the same for spontaneous labor (aRR 1.191, 95% CI 1.010–1.405) and SGA infants (aRR 0.832, 95% CI 0.703–0.984), but not for other outcomes.

**Table 4:** Multiple logistic regression analysis on HBV carriage on pregnancy outcome. Result expressed in *P* value, relative risk (95% confidence intervals). Significant results shown in bold

Pregnancy outcome	Model One	Model Two
Hypertensive pregnancy	0.481, 0.914 (0.713–1.173)	0.349, 0.888 (0.692–1.139)
Gestational hypertension	0.432, 0.851 (0.569–1.273)	0.466, 0.861 (0.576–1.288)
Pre-eclampsia	0.800, 0.961 (0.705–1.310)	0.554, 0.910 (0.667–1.242)
Gestational diabetes	0.090, 0.882 (0.762–1.020)	0.168, 0.902 (0.780–1.044)
Placenta previa	0.110, 0.760 (0.543–1.064)	0.068, 0.731 (0.522–1.023)
Preterm birth < 34 weeks	0.509, 0.885 (0.616–1.271)	0.274, 0.816 (0.568–1.174)
Preterm birth 34- < 37 weeks	0.508, 1.072 (0.872–1.319)	0.816, 1.025 (0.833–1.261)
Spontaneous labor	**0.014, 1.231 (1.044–1.451)**	**0.038, 1.191 (1.010–1.405)**
Large-for-age infants	0.472, 1.068 (0.893–1.277)	0.524, 1.060 (0.886–1.268)
Small-for-age infants	**0.046, 0.842 (0.712–0.997)**	**0.032, 0.832 (0.703–0.984)**
Neonatal death	0.723, 0.873 (0.410–1.855)	0.564, 0.801 (0.376–1.704)

Model one – adjusting for the effects of nulliparity status, maternal age ≥ 35 years, high body mass index, previous miscarriage, abortion and cesarean section

Model two – adjusting for the above factors plus tertiary eduication, and conception by assisted reproduction rtechnology

## Discussion

In this study, the prevalence of maternal HBV infection among women carrying singleton pregnancies resulting in a delivery was 3.3%, which was even lower than the lower range of 5% in the Chinese population reported previously [29]. One reason could be that we had not included women with early pregnancy loss, infertile women, as well as those with high risk profiles who were unlikely to have come under our care in the study cohort. In this way, our study subjects represented the “healthier group” of women in our population for analysis of the effect of HBV infection on obstetric outcome, which is the major concern for our obstetric service, with minimal influence by other confounding factors which are unrelated to HBV infection or by outcomes with implications for the surviving offspring. Nevertheless, it is also possible that in the multi-ethnic society in Kunming, the prevalence of maternal chronic HBV infection is actually lower than that in other regions in China where the majority of the population belongs to the Han ethnicity. Unfortunately our database did not include information on the ethnic group of each woman so that it was not possible to further analyse the prevalence of HBV infection according to each ethnic minority, and future studies are necessary in order to elucidate this aspect.

Our results have demonstrated that in our obstetric population with an intermediate endemic city of 3.3% for HBV infection, HBV status did not exert any significant adverse effect on obstetric outcome. This was largely in line with studies conducted in western societies [8–12] and another in China [35], while the most recently reported prospective cohort study in China found only increased intrahepatic cholestasis, premature rupture of the membranes (but not PTB), and LGA infants [36]. Thus it appears that maternal chronic HBV infection would at most exert minimal or no adverse effects on pregnancy outcome, and at the same time even enhancing fetal growth which could manifest as reduced SGA [9, 12] or increased LGA and macrosomic infants [13–15, 36]. Furthermore, there was a significant independent increase in spontaneous labor, which would in turn reduce the need for intervention such as labor induction. Infants of LBW, even when born at term, have increased risk of hypothermia, a contributing factor to infant mortality [37]. In addition, infants in the lower tertiles of ponderal index have a 3.8-times higher risk of mortality from day 8 to day 365 and a 2.5-times higher risk of hospitalization, compared with infants of greater ponderal indices [38]. Defining in-utero growth impairment by referring to birthweight percentile at birth instead of simply by LBW is more sensitive and previse, and the consistently demonstrable reduction in SGA infants on multivariate analysis in this study would indicate that normal fetal growth is assured instead of increasing fetal overgrowth with consequent birth trauma and other adverse outcomes. Maternal HBV infection was also associated with increased spontaneous labor, thus reducing the need for labor induction which can increase the risk of bilateral spastic cerebral palsy in the infants [39]. Taken together, these outcomes can be seen as subtle features of the “reproductive advantage” conferred by chronic HBV infection on pregnant women [40].

The strengths of our study are the large cohort size, the single center nature with a universal screening policy, uniform approach in antenatal care, and standard management protocols, which would have minimized missing information while ensuring minimal variations in obstetric management which could have influenced the results. However, there are limitations which included the retrospective design and nature of the dataset that did not allow us to ascertain the claimed ethnicity of individual mothers under our care, or to define accurately the age at and activity of HBV infection, and we had no information on other HBV markers such as HBV DNA levels, so that we could not elucidate whether ethnic minorities or HBV activity could have influenced some of the obstetric outcomes within the group of HBV-infected pregnant women. Finally we do not have the resources to follow-up both the mothers and their offsprings so that we cannot ascertain their longer-term outcome. Nevertheless, given the very similar outcome between women with and without HBV infection, any impact of confounding factors such as ethnic minorities or HBV activity would most likely have negligible clinical effects on pregnancy in these women.

In conclusion, the prevalence of maternal HBV infection in our multiethnic city Kunming is 3.3%, which was around the lower range determined in the Chinese population. We found no association between maternal HBV infection with adverse pregnancy outcome, but there was increased spontaneous labor and reduced SGA infants, outcomes which would enhance survival of any offspring infected at birth, thus in turn perpetuates HBV infection through successive generations and maintains its high prevalence in the Chinese population.

## Data Availability

The datasets generated and analyzed during the current study are not publicly available due to the fact that it contains personal information, but are available as the de-identified format from the corresponding author on reasonable request.

## Abbreviations

HBV: Hepatitis B virus
HBsAg: Hepatitis B surface antigen
SGA: Small-for-gestational age
LGA: Large-for-gestational age
PTB: Preterm birth
PHD: Pregnancy hypertensive disorders
GDM: Gestational diabetes mellitus
LBW: Low-birthweight
IUFD: Intrauterine fetal death
SB: Stillbirth
CS: Cesarean section
ART: Assisted reproduction technology
AMA: Advanced maternal age
BMI: Body mass index
ICU: Intensive care unit
SD: Standard deviation
Arr: Adjusted relative risk
